# Graphene-based nanomaterials for stimuli-sensitive controlled delivery of therapeutic molecules

**DOI:** 10.3389/fbioe.2023.1129768

**Published:** 2023-02-09

**Authors:** Elnaz Khakpour, Saba Salehi, Seyed Morteza Naghib, Sadegh Ghorbanzadeh, Wei Zhang

**Affiliations:** ^1^ Nanotechnology Department, School of Advanced Technologies, Iran University of Science and Technology and Biomaterials and Tissue Engineering Department, Breast Cancer Research Center, Motamed Cancer Institute, IUST, ACECR, Tehran, Iran; ^2^ State Key Laboratory of Structure Analysis for Industrial Equipment, Department of Engineering Mechanics, Dalian University of Technology, Dalian, China

**Keywords:** graphene, nanomaterials, stimuli-sensitive, smart drug delivery, endogenous, exogenous

## Abstract

Stimuli-responsive drug delivery has attracted tremendous attention in the past decades. It provides a spatial- and temporal-controlled release in response to different triggers, thus enabling highly efficient drug delivery and minimizing drug side effects. Graphene-based nanomaterials have been broadly explored, and they show great potential in smart drug delivery due to their stimuli-responsive behavior and high loading capacity for an extended range of drug molecules. These characteristics are a result of high surface area, mechanical stability and chemical stability, and excellent optical, electrical, and thermal properties. Their great and infinite functionalization potential also allows them to be integrated into several types of polymers, macromolecules, or other nanoparticles, leading to the fabrication of novel nanocarriers with enhanced biocompatibility and trigger-sensitive properties. Thus, numerous studies have been dedicated to graphene modification and functionalization. In the current review, we introduce graphene derivatives and different graphene-based nanomaterials utilized in drug delivery and discuss the most important advances in their functionalization and modification. Also, their potential and progress in an intelligent drug release in response to different types of stimuli either endogenous (pH, redox conditions, and reactive oxygen species (ROS)) or exogenous (temperature, near-infrared (NIR) radiation, and electric field) will be debated.

## Introduction

**FIGURE 1:**
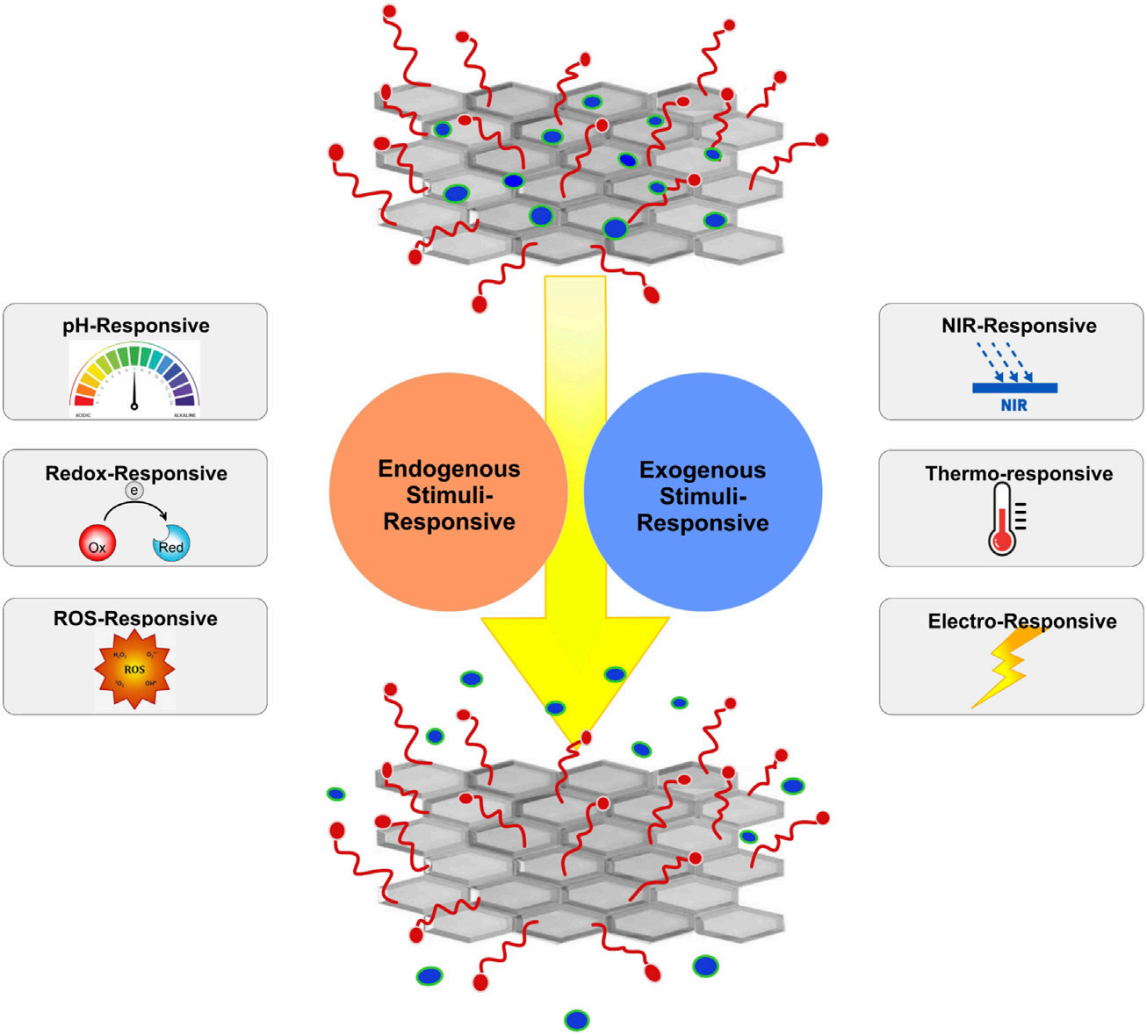
Schematic representation of stimuli-sensitive nanomaterials for the controlled delivery of therapeutic molecules.

Conventional drug delivery methods usually lead to unwanted side effects due to high drug concentrations inserted into the body. Advances in pharmaceutics and material science have led to the invention of controllable drug delivery systems that can minimize toxicity and decrease therapeutic costs. They can load and selectively release a controlled dosage of drug molecules in a specific targeted site, improving the efficiency of therapeutic agents (Bawa et al., 2009; Mura et al., 2013; Chen et al., 2016). However, they only provide a constant release rate and are not adaptable to physiological body conditions (Kost and Langer, 2012). More recently, stimuli-responsive drug delivery systems, being capable of recognizing and reacting to their microenvironment, have provided on-demand drug delivery (Mura et al., 2013). In stimuli-responsive drug delivery systems, a trigger is employed to selectively separate drug molecules from their carrier, thus mimicking the *in vivo* pulsatile release of several types of physiological chemicals, such as hormones, like estrogen and insulin (Murdan, 2003; Raza et al., 2019). The trigger can either be an internal biological inducing factor, resulting from a specific pathological change that is known as an endogenous stimulus, or a physical external factor inserted from outside of the body that is called an exogenous stimulus (Raza et al., 2019). An endogenous stimulus can be pH, redox conditions, or reactive oxygen species (ROS) (Figure 1). Exogenous stimuli include NIR radiation, temperature, and an electric field (Xie et al., 2022).

Nanotechnology plays a crucial role in such smart drug delivery by enabling the synthesis of ideal drug carriers (Chamundeeswari et al., 2019). The exceptional physicochemical properties of nanocarriers allow them to carry high loadings of drugs and deliver them to the targeted tissue with high efficiency (Hossen et al., 2019). Stimuli-responsive nanocarriers are nanomaterial-based compounds than can be designed specifically to release their therapeutic loading upon a particular biochemical, chemical, or physical stimulus (Kamaly et al., 2016). An ideal nanosystem must inherently contain specific targeting functional groups, trigger an explicit biological response, and be detectable. Carbon nanostructures such as graphene and carbon nanotubes (CNTs), due to having rich functionalization potential, can coordinate the fabrication of interesting nanovectors for the targeted delivery of drugs. Graphene with a biocompatible coating and small size appears to not be obviously toxic to animals in a reasonable dose range, as revealed by a number of different studies (Mendes et al., 2013).

### Graphene-based nanomaterials for drug delivery

Graphene is a two-dimensional (2D) carbon allotrope with a hexagonal honeycomb crystal structure (Yu et al., 2020; Patil et al., 2021). Each carbon atom in a graphene monolayer builds a strong sigma (σ) bond with three adjacent carbon atoms. This covalent bond has a short length of ∼1.42 Å that gives graphene an exceptionally strong structure. On the other hand, the presence of free π electrons provides an interlayer binding through weak van der Waals interactions that lead to graphene’s flexibility. Free π electrons can also act as reactive sites that enable graphene to undergo unique surface reactions (Goenka et al., 2014; Yang et al., 2018). Emerging trends show that graphene-based nanomaterials develop unique properties and can be employed for biomedical applications, particularly in drug delivery and tissue engineering (Goenka et al., 2014). Graphene has been recognized as an efficient carrier for an extended range of drug molecules due to its mechanical and chemical stability, the high surface area of its planar structure, great loading potential, and excellent optical, electrical, and thermal properties. Additionally, it has the ability to react to several stimuli such as electric and magnetic fields, pH, temperature, and sound which makes it an ideal candidate for stimuli-responsive drug delivery (Sun et al., 2008; Liu et al., 2011; Bai and Husseini, 2019; Patil et al., 2021). Graphene usage in drug delivery applications was, for the first time, reported by the Dai research group in 2008. They utilized graphene as an effective drug carrier for cancer therapy, which opened the path for its extensive research later on, as a promising material in drug delivery (Song et al., 2020). However, there are still some challenges associated with graphene’s drug loading and release. For example, graphene shows cytotoxicity in biological solutions due to its aggregation. On the other hand, as a strong yet flexible carbon backbone, graphene shows fundamentally unlimited potential for its modification or functionalization (Zhu et al., 2010). Biological modification promotes graphene by enhancing its solubility, selectivity, stability, and biocompatibility (Wang et al., 2011). This will be explained in detail in the modification sector.

### Graphene oxide (GO)

Graphene oxide (GO) is a graphene derivative with a 2D atomic layer, composed of sp2 carbon and sp3 carbon together with oxygen functionalities such as epoxide, hydroxyl, and carboxylic groups. The hexagonal carbon structures and these functional groups lead to versatile surface chemistry and make it possible to form covalent and non-covalent bonds. Rich surface chemistry makes GO more popular than pristine graphene in biomedical applications such as drug delivery. Furthermore, in comparison with graphene, which is insoluble in biological solutions and tends to agglomerate, GO has excellent water solubility (Yang et al., 2015; Kim et al., 2016). Free π electrons in unmodified areas of GO provide hydrophobic regions which are suitable for loading hydrophobic drugs through van der Waals forces (Ghawanmeh et al., 2019). For example, when added to hydrogels, GO’s physical crosslinking enhances the loading capacity of hydrophobic drugs and also improves the stimuli-responsive properties (Chen et al., 2018; Olate-Moya and Palza, 2022).

### Graphene quantum dots (GQDs)

GQDs, as a new generation of carbon-based nanomaterials, have recently displayed great potential in nanomedicine and drug delivery. GQDs have typical dimensions of up to a few nanometers and circular or elliptical shapes (Henna and Pramod, 2020). The active groups present on the GQD surface, similar to graphene and GO, enable them to be conjugated to other molecules, hence making them ideal nanocarriers to simultaneously track and treat the diseased cells. Research studies show that GQD can efficiently prompt the nuclear accumulation of drugs, such as cisplatin and doxorubicin (DOX) (Yang et al., 2015). Additionally, their small size and good biocompatibility serve as favorable properties required for being drug delivery carriers (Zheng et al., 2015). GQDs have also demonstrated therapeutic effects in several diseases like Parkinson’s disease, Alzheimer’s disease, and diabetes in addition to being antibacterial. They also provide drug delivery across the blood–brain barrier (Henna and Pramod, 2020).

### Graphene derivative modification for drug delivery

Numerous studies have been dedicated to graphene modification and functionalization. Graphene and its derivatives can be modified by both covalent functionalization and non-covalent functionalization. GO and rGO, due to having oxygen groups and highly active defects, are more popular than pristine graphene for their modification goals (Zhang et al., 2017). Novel nanohybrids have been developed by conjugating graphene derivatives to different types of materials such as biomacromolecules, polymers, and other nanoparticles to produce unique graphene-based nanocarriers for biocompatible and more effective drug delivery (Wang et al., 2017).

### Modification with polymers

The functionalization of graphene derivatives by suitable polymers enhances graphene’s biocompatibility, solubility, stability, and *in vivo* circulation times. Polyethylene glycol (PEG) is the most extensively researched biocompatible polymer for graphene modification. Its uptake by the reticuloendothelial system is low, and its functionalization on graphene sheets leads to high aqueous solubility and physiological stability, as well as reduced toxicity (Charmi et al., 2019; Sattari et al., 2021). For instance, Charmi et al. (2019) PEGylated GO using the EDC/NHS catalyst *via* esterification bonding, and the fabricated nanohybrid provided a 4.5% loading of curcumin, which is an anticancer drug. The nanocarrier’s ability to pursue pH-responsive delivery was also indicated both *in vitro* and *in vivo*. The phagocytic activity was delayed in blood circulation due to the surface charges, so biocompatibility was confirmed (Charmi et al., 2019). Hyaluronic acid (HA), which is a linear hydrophilic macromolecular polymer, also has been used to modify graphene nanocarriers by several researchers. In a study, HA was conjugated to graphene *via* H-bonding formation between the epoxy groups in GO and the amine groups of HA. The resulting HA−GO nanohybrid was used for targeted and pH-responsive delivery of DOX in certain cancer cells, and it was demonstrated that HA decoration, similar to PEG, improved the solubility and physiological stability of GO, as well as its loading capacity for DOX. Moreover, HA functions as an active targeting moiety to recognize the transmembrane glycoprotein CD44 receptor, which is overexpressed on surfaces of various tumor cells (Song et al., 2014). Furthermore, several research studies have been dedicated to the integration of hydrogels onto graphene in order to enhance drug loading and release. Hydrogels, which are a biocompatible 3D framework of hydrophilic polymers, have been widely used in controlled drug release as they swell in water, and their gel structure changes under different environmental conditions. They can also preserve drugs from the enzymes and acidic environment in the stomach (Tao et al., 2012; Sun et al., 2019). Although hydrogels are suitable carriers for water-soluble drugs such as peptides and proteins, they cannot load efficient amounts of hydrophilic compounds. By adding graphene to such materials, efficient loading capacity for hydrophobic drugs can be provided (Leganés et al., 2020). Polyethylenimine (PEI), chitosan, dendrimers, and hyper-branched polymers are other common polymers used as graphene modifiers for targeted drug delivery (Sattari et al., 2021).

### Modification with biomacromolecules

Biomacromolecules, such as proteins, DNA, and peptides, have attracted so much attention in drug delivery because of their rich functions, biocompatibility, and stability in the body environment. It has been indicated that the conjugation of such macromolecules to graphene derivatives can result in efficient nanocarriers for controlled drug delivery (Wang et al., 2017). For example, Mo et al. (2015) conjugated graphene with an adenosine-50 triphosphate (ATP) aptamer and two single-stranded DNA molecules, which were ATP-responsive. The graphene-DNA crosslinked hybrid inhibited the ATP-responsive release of DOX from GO nanosheets to cancer cells effectively. The high loading capacity of DOX and the site-specific drug release were achieved (Mo et al., 2015). Sima et al. (2020) also functionalized GO nanocolloids by non-covalent conjugation of bovine serum albumin (BSA) protein onto the GO surface to minimize graphene cytotoxicity and as a carrier for anticancer drugs (Sima et al., 2020). Recently, Muzi functionalized GO with hen egg lysozyme (HEL) through a simple non-covalent conjugation method and used the nanocarrier for the selective targeting of B lymphocytes in autoimmune diseases. It was suggested that the same process could be used for other proteins or peptides capable of targeting certain new B cells (Muzi et al., 2021).

### Modification with nanoparticles

In numerous studies, a variety of inorganic nanoparticles have been incorporated into the derivative surface of graphene, providing superior drug delivery. Moreover, the optical and magnetic characteristics of nanoparticles can be used to enable external stimuli-responsive drug delivery and bioimaging. The functional groups and abundant structural defects of GO and rGO offer the benefit of conjugation to nanoparticles, including silica, Au, Ag, Ni, Pt, and Fe_3_O_4_ (Liu et al., 2013).

Mesoporous silica nanoparticles (MSNs), owing to their tunable porosity, large specific surface area, high loading capacity, good biocompatibility, and simple conjugation to target ligands for specific cellular recognition, can develop efficient drug delivery systems. Being functionalized onto graphene, its dispensability and cellular uptake would improve, and an enhanced controlled drug release would be obtained (Manzano and Vallet-Regí, 2020). For example, Tran et al. developed a GO-MSN system for the delivery of cisplatin, a chemophotothermal agent, through NIR/pH-responsive release and fluorescent imaging (Tran et al., 2018). Gold nanoparticles have also been known as promising nanoparticles for drug delivery goals, owing to their exceptional biological and physicochemical features, attachment capability to biomolecules *via* the Au–S bond, photothermal effect, monodispersity, low toxicity, and simple fabrication process. Zhang et al. (2018) utilized the NIR-responsive characteristics of both rGO and gold nanorods (AuNRs) to fabricate a NIR stimuli drug delivery system. The boosted photothermal effect between rGO and AuNPs led to superior photothermal conversion efficiency (about 39%); thus, a more rapid drug release was provided. Moreover, the loading capacity and thermal stability considerably improved (Zhang et al., 2018). Mo et al. (2015) incorporated AuNPs onto GO for effective delivery of DOX to HeLa cells for chemotherapy. AuNPs enabled intracellular Raman imaging as well (Ma et al., 2013). More recently, AuNPs were decorated on PEGylated GO by Samadian et al. for pH-sensitive DOX release. GNPs improved the thermal stability of the nanocarrier, and excellent anticancer performance was achieved mostly due to their high drug-loading capability (Samadian et al., 2020). Ag nanoparticles (AgNPs) incorporated in GO can also enhance the drug loading and release behavior. Rahmani et al. (2022) fabricated polyvinyl alcohol (PVA)-GO-Ag nanofibers loaded with curcumin by electrospinning. It was used for wound healing and was indicated to have a significant inhibitory effect on bacterial growth. Drug loading and encapsulation efficiency rose to 56% and 86%, respectively, owing to the presence of AgNPs. Curcumin release increased in the acidic microenvironment, and also, the growth and proliferation of cells cultured on nanofibers accelerated (Rahmani et al., 2022).

### Graphene-based nanocarriers for stimuli-responsive drug delivery

#### Endogenous stimuli-responsive drug delivery

##### pH-sensitive drug delivery

Different tissues and cellular parts of the body have distinct pH levels that can operate as a stimulus in pH-sensitive drug delivery systems (Zhu and Chen, 2015). In certain abnormal physiological conditions such as cancer, inflammation, and infection, substantial variations of pH have been recognized at the diseased sites. For example, in tumor tissue, glycolysis occurs at high rates, and lactic acid accumulates due to the rapid proliferation of tumor cells and nutrient deficiency. This leads to a notable pH decrease in the tumor microenvironment (5.5–6.8) in comparison with blood and normal tissues (≈7.4), which can act as an endogenous stimulus for acid-sensitive drug delivery systems. In acidic environments, hydrophobic drug molecules like doxorubicin undergo protonation, and hence, the π–π stacking and hydrophobic interactions with the graphene surface weaken, which leads to a pH-responsive release of the drug (Zhu and Chen, 2015; Kamaly et al., 2016; Yang et al., 2016). In 2008, Yang et al. (2016) loaded doxorubicin, an anticancer drug, onto GO for the first time. GO and DOX were mixed in an aqueous solution under mild sonication, and as a result, a high loading of DOX on GO and a strongly pH-dependent drug release was achieved. The high drug loading was attributed to hydrophobic interactions and *π*–*π* stacking between GO and the quinone part of DOX. Furthermore, hydrogen bonding formed between the -NH_2_ and -OH groups on DOX, and -COOH and -OH groups on GO sheets. It was shown that this strong bonding in neutral conditions is responsible for DOX release in acidic and basic environments (Yang et al., 2008). Kavitha et al. (2013) also loaded GO with pH-sensitive poly(2-(diethylamino) ethyl methacrylate) (PDEA) *via* covalent bonding and fabricated a nanocarrier for camptothecin (CPT), a water-insoluble drug, which was attached to GO-PDEA through by π–π stacking and hydrophobic interactions. The nanocarrier released the drug in the lower pH of the tumor environment (pH = 5.5), while no release happened in neutral and basic conditions (Kavitha et al., 2013). In a more recent study, Boddu et al. (2022) developed highly tunable pH-responsive rGO-embedded chitosan beads for the co-delivery of curcumin and 5-fluorouracil and an effective function against MCF7 cells; so, it resulted in the intrinsic anticancer capability (Boddu et al., 2022).

##### Redox-responsive drug delivery

Disulfide bonds (–S-S–) can play a key role in drug delivery devices since they are highly sensitive to redox conditions. They rapidly break inside cells, where the environment is a reducing one due to the high concentration of glutathione (GSH), while the oxidizing extracellular environment provides them long-term stability (Bauhuber et al., 2009). More importantly, GSH concentration in cancer cells is at least four times more than that of normal cells, which causes a bigger intracellular and extracellular redox gradient, leading to efficient drug release in tumor cells (Wang et al., 2016). Research studies have shown that redox-sensitive surface modification of GO with the help of disulfide bonds would enable controllable drug release in a reducing environment (Yang et al., 2016). For example, Kim et al. (2018) conjugated graphene oxide nanoparticles (GONPs) with methoxy poly(ethylene glycol) (MePEG) to fabricate a redox-responsive drug delivery system. They used it to release chlorin e6 (Ce6), a therapeutic compound for cholangiocarcinoma (CCA) cell treatment. GONPs were found to cause a faster release in the presence of glutathione, representing their redox-responsive properties. They also led to a considerably higher amount of drug uptake and ROS yield in the cells, in comparison with Ce6 itself. Moreover, GONPs well accumulated in tumor tissue, while Ce6 itself is mostly gathered in the liver (Kim et al., 2018). It was also demonstrated that the biodegradability of GO could be adjusted by its redox-sensitive surface coating. The Li research group found out that when GO was coated with macromolecules like PEG to fabricate a biocompatible device, it could not be considerably degraded during enzyme-induced oxidization. Hence, they utilized cleavable disulfide bonds for conjugating PEG to GO. The obtained GO-SS-PEG was shown to become considerably degradable. Thus, it was discovered that the redox-responsive surface coating of GO would result in both intelligent drug delivery and tunable biodegradation behaviors (Li et al., 2014).

##### ROS-responsive drug delivery

Reactive oxygen species such as singlet oxygen (^1^O_2_) superoxide (O_2_^−^), hydroxyl radical (·OH), hydrogen peroxide (H_2_O_2_), and hypochlorite ion (OCl^−^) are oxygen ions and free radicals with high reactivity. ROS is produced at low levels in a healthy body and is responsible for adjusting cell signaling and proliferation. However, elevated ROS levels inflict harm on proteins, lipids, and DNA (Trachootham et al., 2009; Saravanakumar et al., 2017). Aging and many pathological conditions such as cancer, inflammation, and atherosclerosis are associated with ROS over-production in the body (Yang et al., 2016). Typical injection of oxidation-responsive nanoparticles and hydrogels often results in rapid degradation or low bioavailability in ROS conditions and would not work efficiently. Wu et al. (2022) could fabricate a ROS-responsive nanofiber membrane using rGO as a nanocarrier and PEGDA-EDT as a ROS-sensitive motif for fucoxanthin (Fx) delivery. In an H_2_O_2_ environment, the nanofiber membrane showed a sustained and long-term Fx release behavior and low toxicity (Wu et al., 2022).

#### Exogenous stimuli-responsive drug delivery

##### NIR-responsive drug delivery

**FIGURE 2:**
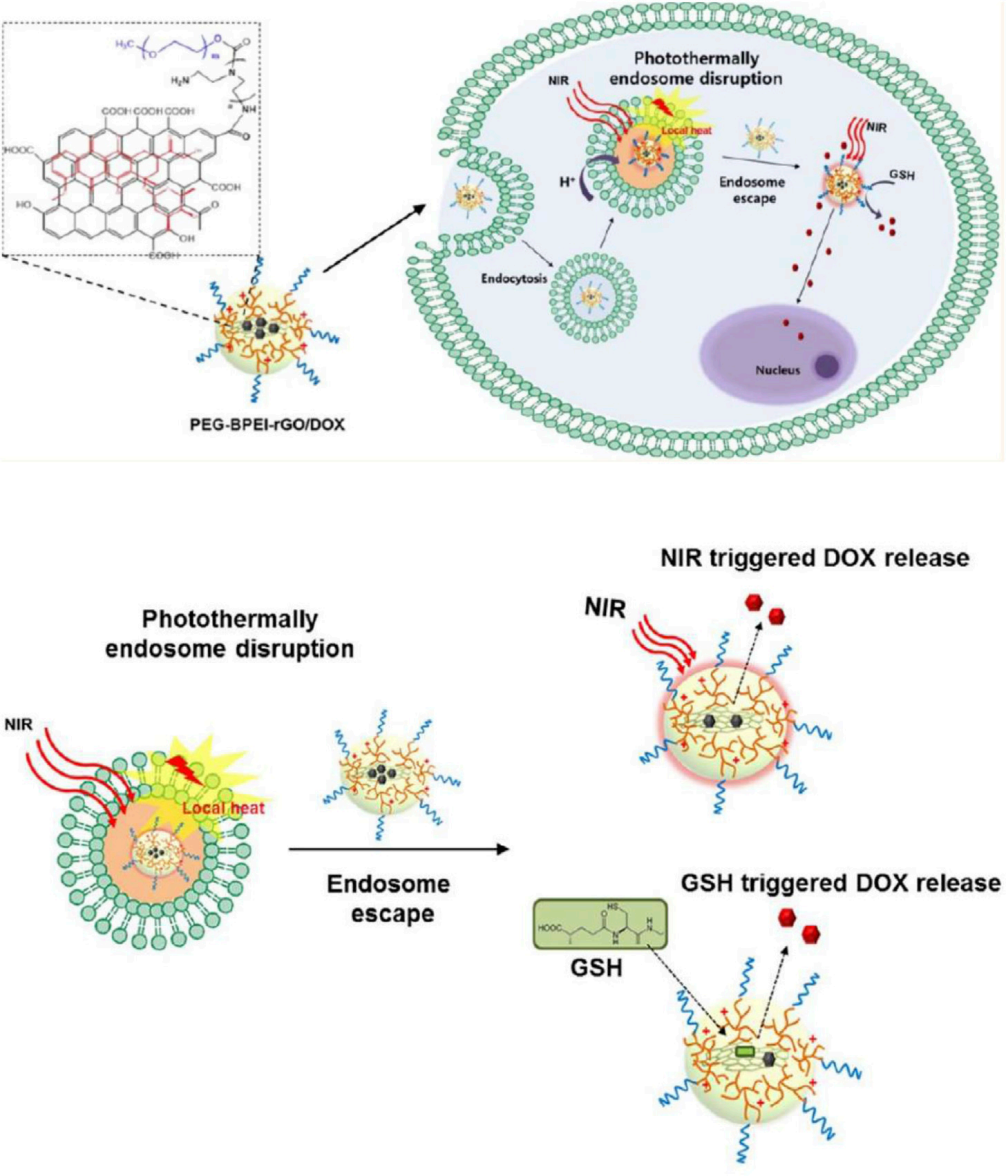
Schematic representation of the mechanism of cytosolic drug release by near-infrared (NIR) radiation and glutathione (GSH) after photothermally induced endosome disruption, reprinted with permission from Kim et al. (2013), copyright 2013, American Chemical Society.

Near-infrared (NIR) radiation has been verified to be a capable method for photothermal cancer treatment. Having a wavelength between 650 and 900 nm, NIR radiation has minimal body absorbance, while it could easily penetrate tissue for micrometers to centimeters, allowing photothermal drug release (Timko et al., 2010). Carbon-based nanomaterials are popular agents in NIR-responsive drug delivery systems due to high optical absorbance in the NIR range (Song et al., 2016). Light-responsive drug delivery is generally achieved through either photothermal or photochemical reactions. In photothermally induced drug delivery, nanocarriers go under light-heat transformation when they are exposed to an optical trigger and the obtained increasing local temperature would lead to drug release. By contrast, photochemically triggered drug delivery is achieved by photo-induced cleavage or reactions, or photo-dimerization or isomerization due to the presence of groups or bonds sensitive to light (Xiao et al., 2012). Graphene oxide has been reported to have great photothermal properties. In 2013, Kim et al. developed a photothermal-responsive cytosolic nanocarrier by functionalizing reduced graphene oxide to PEG-BPEIrGO for DOX delivery into cancer cells. First, endosome disruption occurred by photothermal induction, and then, the drug was released by NIR irradiation. Additionally, the presence of GSH induced more rapid DOX release by deteriorating the π–π stacking and non-covalent hydrophobic interactions of GO. The reported nanotemplate had a greater loading capacity for DOX and also higher water stability than PEG-BPEI-GO which contained unreduced GO due to hydrophobic interactions and π–π stacking. After cellular uptake and before lysosomal degradation, the nanocarrier successfully escaped from the endosome by photothermally induced endosomal disruption of rGO and the proton sponge effect of BPEI. Subsequently, the efficient GSH-mediated release of DOX into the cytosol was observed (Figure 2) (Kim et al., 2013).

Wang et al. (2021) reported the synthesis of chitosan hydrogel films loaded with reduced graphene oxide (CS/rGO) as NIR light-responsive nanocarriers for local delivery of teriparatide, a drug for osteoporosis treatment. The biomimetic pulsatile release was achieved through photothermal conversion for osteoporotic bone regeneration in rats. The results showed that by increasing hydrogel’s rGO content, the teriparatide loading capacity rose, and when rGO content reached 0.7%, 85% drug loading was obtained. Also, by dedicating more time to NIR irradiation, more drug amounts were released. Furthermore, more blood vessels were noticed among the regenerated bone and the defect’s center. The fabricated system provided a new approach to repairing osteoporotic bone defects by keeping more amounts of the drug in the defective area without any systemic side effects. It has also been reported that GO can photochemically respond to NIR radiation (Wang et al., 2021). For example, He et al. fabricated a photochemically induced NIR-responsive nanocarrier (MnCO-GO) by captivating Mn-carbonyl CORMs in a small GO nanosheet. The nanomedicine was demonstrated to be highly controllable and NIR-responsive/sensitive to CO release from trapped CORMs. When it was subjected to NIR radiation, GO absorbed the light and converted the photons to active electrons. The electrons in the GO sheet were transferred to Mn-carbonyl molecules, and contesting 3d orbitals of Mn with carbonyls resulted in CO separation from Mn (He et al., 2015).

##### Thermo-responsive drug delivery

Thermo-responsive hydrogels are relatively the most researched responsive hydrogel systems because they show exclusive properties in controlled drug delivery systems. Among them, poly (N-isopropylacrylamide) (PNIPAAm)-based hydrogels have been widely used in thermo-responsive drug delivery since they exhibit a reversible phase transition temperature of about 32°C that is close to the body temperature. Yet, they face challenges including low biocompatibility, low mechanical strength, and slow response that limit their feasibility. To prompt their response, nanohydrogels have been developed. It is also essential to avoid using chemical crosslinkers in synthesizing hydrogels since it results in their toxicity. Functionalizing GO with many biocompatible polymers such as chitosan, polyethylene glycol, and PNIPAAm has benefited gene and tumor drug delivery. Sattari et al. fabricated thermo-responsive GO-based hydrogels through *in situ* polymerization of NIPAAm in a GO/modified matrix as a non-toxic hydrogel crosslinker. They showed that by increasing the GO content in a hydrogel composite, the hydrogel’s phase transition temperature, thermal stability, and internal network crosslinking increased (Sattari et al., 2017).

Havanur and JagadeeshBabu (2018) synthesized a novel intelligent DOX carrier using the poly (N,N-diethyl acrylamide) (PDEA) hydrogel, which is a temperature-responsive macroporous polymer. They loaded it with graphene quantum dots to increase its lower critical solution temperature (LCST). It was demonstrated that by loading more GQD content, the hydrogel’s porous structure got more interconnected by having a higher number of smaller pores. It happened due to water molecule crystallization in its swollen state. As a result, the hydrogel’s equilibrium swelling ratio (ESR) increased considerably and led to more rapid water transport, and subsequently caused improved temperature sensitivity. The addition of GODs also decreased DOX cytotoxicity by enhancing the hydrogel’s loading capacity from 70% to 99% (Havanur and JagadeeshBabu, 2018).

A thermo-responsive hydrogel scaffold was also developed by Mauri et al., in 2020, that incorporated pristine few-layer graphene without distortions associated with the oxidation processes. Diclofenac, a non-steroidal drug for the treatment of musculoskeletal and systemic inflammations, was physically adsorbed on the carrier matrix by providing π–π interactions between its twisted phenyl rings and graphene. Additionally, the defects or vacancies of pristine graphene provided van der Waals interaction and hydrophobic interaction, as well as hydrogen bonds upon the energy and polarizability of the bonds and interactions. By increasing the temperature from 25°C (room conditions) to 44°C (hyperthermia treatment conditions) over time, tunable drug release was identified, while a temperature-independent release kinetic was observed in the lattice without few-layer graphene. Thus, it was suggested that graphene’s π-conjugated structure would modify the electrostatic interactions with the diclofenac molecule and promote the thermal response (Mauri et al., 2021).

##### Electro-responsive drug delivery

Electric field, as an exogenous stimulation for intelligent drug delivery, has attracted so much attention. This is mostly because of its simplicity, portability, and low cost so that it can be easily utilized for personalized applications by inducing a low voltage. Using molecules that orient their dipoles under electric fields is necessary for electro-responsive devices. Nevertheless, responsiveness is more commonly obtained by molecules that undergo an electrically induced redox reaction (Zhao et al., 2016). Electro-responsive nanomaterials are generally synthesized by utilizing polyelectrolytes, which have the ability to shrink or swell when subjected to electrical fields. However, most of the polymers, which have been commonly used in drug release systems, lack essential electrical conductivity. It has become possible to overcome this limitation by integrating conducting nanomaterials into polymeric scaffolds. When graphene or its derivatives are incorporated into polymers and hydrogels, unique chemical structures and attractive physiochemical properties of graphene lead to the synthesis of composites that are highly biodegradable and biocompatible in a cellular environment and have great cellular uptake and highly responsive behavior (Cirillo et al., 2016). In 2013, Liu et al. fabricated rGO-based hydrogels for the delivery of lidocaine hydrochloride through the stimulation of an external electric field. Although it was demonstrated that the addition of GO led to a highly controllable and responsive release in the presence of an electrical trigger, large voltages were needed to modulate drug release and this might damage biological tissues (Liu et al., 2012). Servant et al., in 2014, developed an electro-responsive macroporous hydrogel matrix loaded with pristine graphene sheets for *in vivo* pulsatile drug release. The reported hybrid scaffold solved the two major challenges that the prior electro-responsive drug delivery devices had dealt with. First, the resistive heating and the following temperature rise caused by the electric field stimuli were eliminated. Due to the presence of pristine graphene at low concentrations, drug release became possible through short stimulations and at low voltages. Second, the drug release reproducibility was provided between the ON–OFF electrical stimulation upon using low electrical voltages (Servant et al., 2014). By depositing GO into a conductive polymer network, Weaver et al. also achieved an electro-responsive drug delivery device with dosage flexibility, favorable electrical properties, and a high level of temporal control for the delivery of dexamethasone, an anti-inflammatory molecule. They could tune the drug loading content and release profile by lowering the thickness and size of GO nanosheets (Weaver et al., 2014). More recently, Sahoo et al. (2022) developed a novel electro-responsive graphene oxide (GO) nanoparticle system and examined it for *in vitro* simultaneous delivery of aspirin and doxorubicin in MDA-MB 231 breast cancer cells. Dual drug delivery is more effective than utilizing a single drug delivery and leads to lower drug resistance and fewer side effects. The on-demand drug delivery in the presence of external low voltage was remotely controlled by a mobile phone (Du et al., 2020).

## Conclusion

Graphene has been identified to have selective and exceptional drug loading and release characteristics, owing to its high surface area, mechanical and chemical stability, and excellent optical, electrical, and thermal properties. Moreover, it can react to several stimuli such as electric field, pH, and temperature. In this review, graphene-based nanocarriers for smart drug delivery were introduced. Graphene oxide, due to containing oxygen functionalities such as epoxide, hydroxyl, and carboxylic groups, has a more versatile surface chemistry than pristine graphene which makes it soluble in biological solutions and enables its conjugation to several molecules and drugs. Graphene quantum dots also prompt the nuclear accumulation of drugs, and having a reduced size makes them an advantageous carrier for more efficient drug delivery. Additionally, the surface modification of graphene derivatives with appropriate molecules such as polymers, biomacromolecules, and nanoparticles, which can be conjugated by either covalent and/or non-covalent interactions, has been summarized. The addition of these molecules to graphene derivatives reduces their toxicity and enhances biocompatibility, solubility, and stability, thus providing unique graphene-based nanocarriers for biocompatible and more effective drug delivery. Furthermore, response to both internal and external stimuli such as pH gradients, reducing agents, ROS, electric field, temperature, and NIR radiation, which have been researched numerously, was debated.
